# Towards a Cascading Reasoning Framework to Support Responsive Ambient-Intelligent Healthcare Interventions

**DOI:** 10.3390/s18103514

**Published:** 2018-10-18

**Authors:** Mathias De Brouwer, Femke Ongenae, Pieter Bonte, Filip De Turck

**Affiliations:** IDLab, iGent Tower—Department of Information Technology, Ghent University—imec, Technologiepark-Zwijnaarde 15, B-9052 Ghent, Belgium; Femke.Ongenae@UGent.be (F.O.); PieterS.Bonte@UGent.be (P.B.); Filip.DeTurck@UGent.be (F.D.T.)

**Keywords:** pervasive healthcare, cascading reasoning, stream reasoning

## Abstract

In hospitals and smart nursing homes, ambient-intelligent care rooms are equipped with many sensors. They can monitor environmental and body parameters, and detect wearable devices of patients and nurses. Hence, they continuously produce data streams. This offers the opportunity to collect, integrate and interpret this data in a context-aware manner, with a focus on reactivity and autonomy. However, doing this in real time on huge data streams is a challenging task. In this context, cascading reasoning is an emerging research approach that exploits the trade-off between reasoning complexity and data velocity by constructing a processing hierarchy of reasoners. Therefore, a cascading reasoning framework is proposed in this paper. A generic architecture is presented allowing to create a pipeline of reasoning components hosted locally, in the edge of the network, and in the cloud. The architecture is implemented on a pervasive health use case, where medically diagnosed patients are constantly monitored, and alarming situations can be detected and reacted upon in a context-aware manner. A performance evaluation shows that the total system latency is mostly lower than 5 s, allowing for responsive intervention by a nurse in alarming situations. Using the evaluation results, the benefits of cascading reasoning for healthcare are analyzed.

## 1. Background

### 1.1. Introduction

The ultimate ambient-intelligent care rooms of the future in smart hospitals or smart nursing homes consist of a wide range of Internet of Things (IoT) enabled devices equipped with a plethora of sensors, which constantly generate data [1,2]. Wireless Sensor Networks (WSNs) can be used to monitor environmental parameters, such as light intensity and sound, and Body Area Networks (BANs) can monitor vital body parameters, such as heart rate, blood pressure or body temperature. Other IoT-enabled devices allow for performing indoor positioning, to detect when doors or windows are opened, or to discover if a patient is lying in bed or sitting in a couch. Intelligent smart home IoT devices can be used to take control of and automate the lighting, window blindings, heating, ventilation and air conditioning (HVAC), and more. Moreover, domain and background knowledge contains information on diseases, medical symptoms, the patients’ Electronic Health Record (EHR), and much more. The advantage of the IoT is that the data streams originating from the various sensors and devices can be combined with this knowledge to derive new knowledge about the environment and the patient’s current condition [3]. This enables devices to achieve situation- and context-awareness, and enables better support of the nursing staff in their activities [4].

Consider the example of a pervasive health context in which a patient suffers from a concussion. Medical domain knowledge states that concussion patients are sensitive to light and sound. This knowledge can be combined with data streams coming from the light and sound sensors in the patient’s room, to derive when an alarming situation occurs, i.e., when the patient is in his room and certain light or sound thresholds are crossed. When such an alarming situation is detected, automatic action can be taken, such as autonomously dimming the lights or alerting a caregiver. This can help to increase the comfort of both the patients and nurses, and help nurses to operate more efficiently.

By 2020, 20 to 30 billion IoT devices are forecasted to be in use worldwide within healthcare [5]. The data streams generated by these IoT devices are not only voluminous, but are also a heterogeneous, possibly noisy and incomplete set of time-varying data events [6]. As such, it is a challenging task to integrate, interpret and analyze the data streams on the fly to derive actionable insights.

Semantically enriching the data facilitates the consolidation of these data streams [7]. It imposes a common, machine-interpretable data representation. It also makes the properties of the device and the context in which the data was gathered explicit [7]. Moreover, it enables the integration of these streams with the domain and background knowledge.

Semantic Web technologies, such as the Resource Description Framework (RDF) and the Web Ontology Language (OWL), allow for achieving this semantic enrichment by using ontologies [7]. An ontology is a semantic model that formally describes the concepts in a particular domain, their relationships and attributes [8]. Using an ontology, heterogeneous data can be modeled in a uniform way. Recently, ontologies for the IoT have emerged, such as the Semantic Sensor Network (SSN) ontology [9], which facilitate the enrichment of IoT data. Moreover, prevalent healthcare ontologies exist, such as SNOMED [10] and FHIR [11], which model a lot of medical domain knowledge. By using the Linked Data approach [12], the semantic IoT data can then easily be linked to such domain knowledge and resources described by these and other models. Semantic reasoners, e.g., Hermit [13] and RDFox [14], have been designed to interpret this semantic interconnected data in order to derive useful knowledge [15], i.e., additional new implicit knowledge that can be useful for applications. For example, in the case of the concussion patient, a semantic reasoner can automatically derive that the patient is sensitive to light and sound, and that light and sound sensors in the patient’s room should be monitored. Based on the definitions of alarming situations in the ontology, the reasoner can infer from the data streams when such a situation occurs.

The complexity of semantic reasoning depends on the expressivity of the underlying semantic language, i.e., the expressivity of the ontology [16]. Different sublanguages exist, ranging from RDFS, which supports only simple statements, e.g., class inheritance, to OWL 2 DL, which supports expressive reasoning, e.g., cardinality restrictions on properties of classes. In OWL, it is assumed that any instance of a class may have an arbitrary number (zero or more) of values for a particular property. According to the W3C definition, cardinality constraints can be used to require a minimum number of values for that property, to allow only a specific number of values for that property, or to specify an exact number of values for that property. For example, in healthcare, it could be defined that an Observation is made by exactly 1 Sensor. Other profiles, such as OWL 2 RL, OWL 2 QL and OWL 2 EL, are situated in between RDFS and OWL 2 DL, trading off reasoning expressivity for computational efficiency. More expressive reasoning allows for deriving more interesting information from the streams and transforms it to actionable insights. In healthcare, high expressivity of the reasoner is required. In the example of the concussion patient, alarming situations can have complex definitions, making it impossible for less expressive reasoners to infer their occurrence. For example, an RDFS reasoner would not be able to infer that a patient with a concussion is sensitive to light and sound.

Semantic reasoning over large or complex ontologies is computationally intensive and slow. Hence, it cannot keep up with the velocity of large data streams generated in healthcare to derive real-time knowledge [15]. However, in healthcare, making decisions often is time-critical. For example, alarming situations for a patient should be reacted upon in a responsive manner. In this case, real-time means within a time frame of 5 s. For each situation or use case, real-time can be defined differently. In addition, available resources are limited, making the computational complexity of expressive reasoning for healthcare even a bigger issue. Moreover, when constructing a solution for these problems, privacy management of the patient data is an important consideration [17].

To tackle the issue with performing real-time analysis, two research trends have emerged, being stream reasoning and cascading reasoning. Stream reasoning [15] tries to incorporate semantic reasoning techniques in stream processing techniques. It defines a data stream as a sequence of time-annotated items ordered according to temporal criteria, and studies the application of inference techniques to such streaming data [15]. Cascading reasoning [18] exploits the trade-off between reasoning complexity and data stream velocity by constructing a processing hierarchy of reasoners. Hence, there is the need for a platform using these techniques to solve the issues in smart healthcare.

### 1.2. Objective and Paper Organization

The objective of this paper is the realization of a generic cascading reasoning platform, and the study of its applicability to solve the aforementioned smart healthcare issues. The cascading reasoning platform is implemented in an open flexible way, to make it easily extensible and applicable to different use cases. A Proof-of-Concept (PoC) application is implemented on a use case situated in pervasive healthcare. Using the PoC implementation, the performance of the framework is evaluated, and its advantages and disadvantages are discussed.

The remainder of this paper is organized as follows. Section 2 discusses the related work. In Section 3, the general architecture of the proposed cascading reasoning platform is described. Section 4 and Section 5 address the use case for the PoC and its implementation using the architecture components. Section 6 then describes the evaluation set-up, including the different evaluation scenarios and hardware set-up. Results of this evaluation are presented in Section 7. In Section 8, the evaluation results, advantages and disadvantages of the platform for the PoC use case are further discussed. Finally, Section 9 concludes the main findings and highlights future work.

## 2. Related Work

### 2.1. Stream Reasoning

Data Stream Management Systems (DSMS) and Complex Event Processing (CEP) systems allow to query homogeneous streaming data structured according to a fixed data model [19]. However, in contrast to Semantic Web reasoners, DSMS and CEP systems are not able to deal with heterogeneous data sources and lack support for the integration of domain knowledge in a standardized fashion.

Therefore, stream reasoning [15] has emerged as a challenging research area that focuses on the adoption of semantic reasoning techniques for streaming data. The first prototypes of RDF Stream Processing (RSP) engines [20] mainly focus on stream processing. The most well-known examples of RSP engines are C-SPARQL [21] and CQELS [22], but others also exist, such as EP-SPARQL [23] and SPARQLStream [24]. Because a continuous data stream has no defined ending, a window is placed on top of the data stream. A continuous query is registered once and produces results continuously over time as the streaming data passes through the window. As such, these RSP engines can filter and query a continuous flow of data and can provide real-time answers. Each of these engines has different semantics and is tailored towards different use cases. Other solutions, e.g., Sparkwave [25] and INSTANS [26], use extensions of the RETE algorithm [27] for pattern matching.

As shown in Table 1, all considered RSP engines, except INSTANS, support integration of domain knowledge in the querying process. However, their reasoning capabilities are limited. None of the proposed systems is able to efficiently perform expressive OWL 2 DL reasoning on streaming data, which is often required for complex application domains such as healthcare.

StreamRule [28] is a 2-tier approach, combining stream processing with rule-based non-monotonic Answer Set Programming (ASP) to enable reasoning over data streams. However, ASP is not standardized, meaning no existing healthcare vocabularies can be exploited, in contrast to OWL.

In summary, stream reasoning tries to adopt semantic reasoning techniques for streaming data, but still lacks the possibility to support real-time and expressive reasoning at the same time. The existing available RSP engines aim at filtering and querying of streaming data, but they lack support for complex reasoning. To perform such complex reasoning, traditional semantic reasoners need to be used. However, this complex reasoning is computationally intensive and not capable of handling streaming data.

### 2.2. Cascading Reasoning

The concept of cascading reasoning [29] was proposed to exploit the trade-off between reasoning complexity and data stream velocity. The aim is to construct a processing hierarchy of reasoners. At lower levels, high frequency data streams are filtered with little or no reasoning, to reduce the volume and rate of the data. At higher levels, more complex reasoning is possible, as the change frequency has been further reduced. This approach avoids feeding high frequency streaming data directly to complex reasoning algorithms. In the vision of cascading reasoning, streams are first fed to one or more RSP engines, and then to more expressive semantic reasoners.

The concept of cascading reasoners fits nicely with the current trend in IoT architectures towards Fog computing [30], where the edge is introduced as an intermediate layer between data acquisition and the cloud-based processing layer. The edge allows filtering and aggregation of data, resulting in reduced network congestions, less latency and improved scalability. In addition, it enables to process the data close to its source, which in turn can improve the response time, as it allows to rapidly act on events occurring in the environment. As such, fast and possibly less accurate derivations and actions can be made at the edge of the network. These intermediate results can then be combined and forwarded to the cloud for further, more complex and less time-stringent processing. In this way, more privacy is also enabled. For example, these intermediate results can filter out sensitive data, to avoid sending it over the network.

Recently, much research effort has been put into Fog computing [31,32], and Fog computing frameworks, such as FogFrame [33], are being designed. These frameworks focus on the creation of a dynamic software environment to execute services in a distributed way. They are useful for system deployment, execution and monitoring, but not sufficient to support cascading reasoning. In addition, a generic framework is required that enables cascading reasoning across the IoT fog.

Different distributed semantic reasoning frameworks exist, e.g., DRAGO, LarKC, Marvin and WebPIE. However, they all have limitations in terms of applicability for the IoT [34]. First, they do not consider the heterogeneous nature of an IoT network. In particular, the use of low-end devices and networking aspects are not considered. Both criteria are, however, essential to Fog computing. Second, as they distribute reasoning evenly across nodes, cascading reasoning is not considered. Third, they do not focus on complex reasoning. Hence, these frameworks cannot be used as such.

Recent stream reasoning research also touches the area of Fog computing and devices with limited resources. Various relevant topics are addressed, from publishing RDF streams from smartphones [35], over optimizing semantic reasoning in memory-constrained environments [36], to optimizing the format to exchange and query RDF data in these environments [37]. These results can be useful for a cascading reasoning system, but do not solve the need for a generic cascading reasoning framework.

### 2.3. Frameworks for Healthcare and Ambient Assisted Living

Within healthcare, Ambient Assisted Living (AAL) solutions offer IT products, services and systems that focus on the improvement of an individual’s Quality of Life [38]. Enhanced Living Environments (ELE) include all technological achievements that support AAL environments [39].

Goleva et al. have presented a generic reference architecture for AAL and ELE platforms [40]. This architecture defines an AAL/ELE system as a distributed heterogeneous network. It supports the envisioned AAL as a Service (AALaaS) and ELE as a Service (ELEaaS) [39]. The architecture incorporates the Fog computing principles, by making a distinction between services running in the edge and in the cloud. Locally, sensor networks collect all data, which is transferred through the network. At Fog computing level, the architecture consists of regional components that perform local computation and storage. The goal of this generic reference architecture is to allow data processing to be done at different levels, depending on security, privacy, end-user preferences, technology, legislation, energy requirements etc. It also enables real-time processing for critical data. As such, this generic architecture perfectly fits with the idea of deploying a cascading reasoning system across the IoT fog.

Table 2 compares the most prevalent state-of-the-art AAL frameworks and solutions [41,42,43,44,45,46,47,48,49,50,51] to the solution presented in this work. Many of these approaches are still cloud-based. However, approaches using Fog computing principles, where AAL system components are distributed across heterogeneous devices, are being incorporated more and more. Most existing AAL solutions specifically focus on the incorporation of semantic components in AAL systems. They all make use of reasoning techniques in some way, but stream reasoning is not tackled by any of them. Cascading reasoning is only tackled by one approach, ERMHAN [50], which distributes knowledge and reasoning across two nodes. In summary, there currently does not exist a semantic AAL solution that uses stream reasoning techniques in a cascading fashion across the IoT fog.

### 2.4. Contribution

The contribution of this work is the realization of the vision of cascading reasoning through a framework, using stream reasoning techniques and following the Fog computing principles. Stream reasoning techniques are required in order to infer real-time knowledge and actions from the voluminous and heterogeneous background knowledge and streaming data sources. However, current stream reasoning solutions fail to combine real-time and expressive reasoning. Incorporating them in a cascading fashion is a possible solution. In addition, cascading reasoning and Fog computing principles offer the potential to solve the existing issues in smart healthcare. These concepts have not yet been combined for AAL in previous works, as is shown in Table 2. Therefore, the contribution of this work is to combine them in a framework.

In concrete, the contribution of this work is threefold. First, an architecture is designed for this framework that fits within the generic reference architecture for AAL and ELE platforms [40]. Second, a concrete PoC implementation of this architecture for a specific pervasive healthcare use case is performed. Third, an evaluation of this PoC is conducted using three simulation scenarios.

## 3. Architecture of the Cascading Reasoning Framework

**Figure 1:**
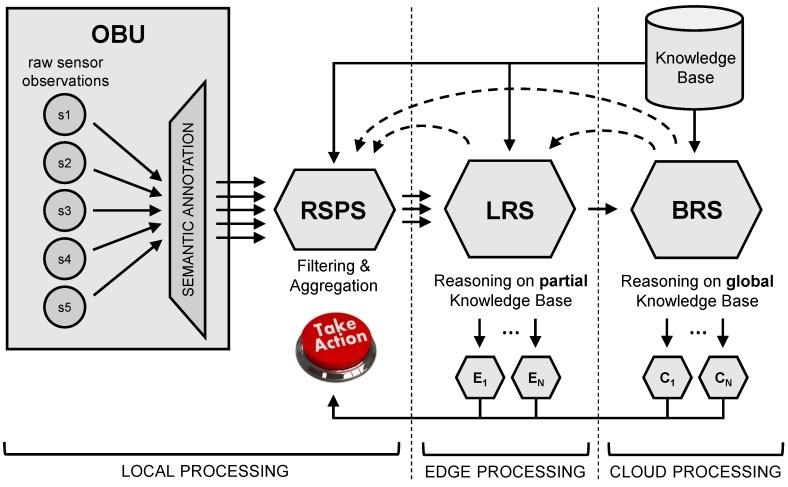
High-level architecture of the proposed cascading reasoning framework. The blocks represent the several components, the arrows indicate how the data flows through these components. The dotted arrows indicate possible feedback loops to preceding components.

The architecture of the generic cascading reasoning framework consists of four main components: an Observation Unit (OBU), an RSP Service (RSPS), a Local Reasoning Service (LRS) and a Back-end Reasoning Service (BRS). Furthermore, a central knowledge base is available. An overview of this architecture is given in Figure 1.

The central knowledge base contains the domain ontologies and static context information. For example, in healthcare, the knowledge base includes information on existing medical domain knowledge and a semantic version of the EHR of patients. The information in the knowledge base can be managed in a centralized database system, which can be an RDF triple store. In this case, the triple store contains mappings of the existing data architecture to the supported ontological formats. In addition, the information can also be managed in a regular database system that supports ontology-based data access (OBDA) [52].

The OBU refers to the infrastructure used to monitor the given environment. This infrastructure can consist of WSNs, BANs and other sensor platforms. The task of the OBU is threefold: (i) capture the raw sensor observations, (ii) semantically annotate these observations and (iii) push the resulting set of RDF triples on its corresponding output RDF data stream. This set of RDF triples should consist of a reference to the sensor producing the observation, the observed value and an associated timestamp.

The RSPS, LRS and BRS components are the stream processing and reasoning components. By configuration, only the relevant parts of the central knowledge base are available on each RSPS and LRS component, as these components typically do not need to know all domain knowledge and/or context information of the full system. On each BRS component, the full central knowledge base is available. Updates to the knowledge bases are coordinated from the BRS component(s).

The RSPS is situated locally and contains an RSP engine. The input of this engine consists of the RDF data streams produced by the OBU, or another RSPS. The task of the RSP engine is to perform some initial local processing of these input data streams, by aggregating and filtering the data according to its relevance. On the RSPS, little to no reasoning is done on the data, depending on the use case. The output of the RSP engine is one or more RDF streams of much lower frequency, containing the interesting and relevant data that is considered for further processing and reasoning.

The LRS is situated in the edge of the network. Here, a local reasoner is running that is capable of performing more expressive reasoning, e.g., OWL 2 RL or OWL 2 DL reasoning. It takes as input the output RDF stream(s) of the RSPS, or another LRS. As the velocity of these streams is typically lower than the original stream, computation time of the reasoning can be reduced. The service has two main responsibilities. First, it can push reasoning results and/or data patterns to another LRS, or a BRS in the cloud, for further processing. Again, the output stream typically is of lower frequency than the input streams. Second, it can also push results to one or more other external components that are capable of performing some first local actions. This allows for fast intervention, before the results are further processed deeper in the network.

The BRS is situated in the cloud. It also consists of an expressive reasoner that has access to the full central knowledge base. Typically, a small number of BRS components exist in the system, compared to several LRS and even more RSPS components. The reasoning performed by the BRS can be much more complex, as it is working on data streams of much lower frequency compared to the local and edge components. This enables to derive and define intelligent and useful insights and actions. These insights and action commands can be forwarded to other BRS or external components, which can then act upon the received information or commands.

In some use cases, it might be useful to provide feedback to preceding components in the chain. This is possible in the current architecture, by the use of feedback loops. This feedback can be seen as messages, e.g., events or queries, in the opposite direction of the normal data flow.

When deploying the architecture, each component in the system should be configured. To this end, the observer concept is used: each component, including external components, can register itself as an observer to an output stream of another component. In this way, the system components can be linked in any possible way. Hence, using the generic architecture, an arbitrary network of components can be constructed and configured. It should be noted that this is a push-based architecture, where the outputs of each component are immediately pushed to the input stream(s) of its observers.

Note that this architecture assumes the following prerequisites: (i) the security of the architecture has been set up and ensured; (ii) no loss of connectivity to the cloud is assumed; (iii) each component runs on a node with at least 1 GB of memory resources; and (iv) each component is available at all times.

**Figure 2:**
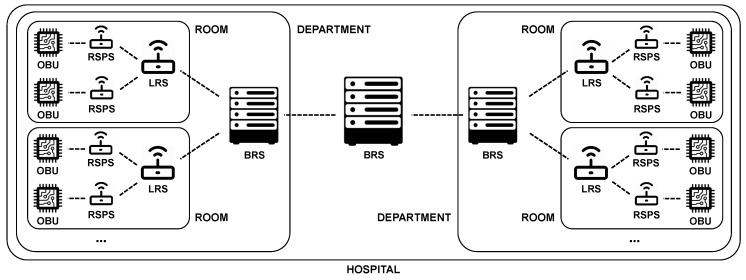
Potential deployment of the architecture of the cascading reasoning platform in a hospital setting. A network of components can be constructed. In this example, there is one Observation Unit (OBU) and RSP Service (RSPS) per patient, one Local Reasoning Service (LRS) per room, one Back-end Reasoning Service (BRS) per department, and another BRS for the full hospital. Potential external components are omitted from this figure.

Figure 2 shows a potential deployment of this architecture in a hospital setting. In this example, there is one OBU and RSPS per patient, one LRS per room, one BRS per department, and another BRS for the full hospital.

## 4. Use Case Description and Set-Up

A PoC has been developed for a use case situated in pervasive healthcare. In this section, this use case is described in detail, followed by a discussion of how the use case has been mapped to the architecture described in Section 3. Moreover, the designed continuous care ontology and the data sources for the use case are discussed. The implementation of the different architecture components is given in Section 5.

### 4.1. Pervasive Health Use Case Description

Consider a use case where a patient Bob is suffering from a concussion and is therefore being hospitalized. The EHR of Bob states that he suffers from a concussion, meaning that direct exposure to light and sound must be avoided. Both Bob’s EHR and this medical domain knowledge are available in the knowledge base. Based on this data, an acceptable level for both light intensity and sound level to which Bob may be exposed to (one of *none*, *low*, *moderate*, *normal* or *high*) can automatically be suggested and added to the knowledge base. These personalized levels can then be adjusted by a doctor or the nursing staff, if required. For Bob, the acceptable level of both light intensity and sound is *low*. For each property, a mapping between the acceptance levels and absolute threshold values is also part of the medical domain knowledge. For example, a *moderate* light intensity level is mapped to a light intensity threshold of 360 lumen, meaning that 360 lumen is the maximum light intensity that a patient with this acceptance level may be exposed to. In Bob’s case, the threshold values for light intensity and sound are 180 lumen and 30 decibels, respectively.

The hospital room where patient Bob is accommodated, is equipped with an OBU. This OBU has multiple sensors, among which a light and sound sensor. The OBU can also detect the presence of people in the room through the presence of a Bluetooth Low Energy (BLE) sensor (beacon). As all patients and nurses in the hospital are wearing a BLE bracelet containing a personal BLE tag, the system is able to discover all relevant people present in the room.

When the observed light intensity or sound values in Bob’s room exceed the thresholds related to the acceptance levels found in his EHR, a *possibly unpleasant situation* for Bob is detected. This situation is called a *symptom*. Possibly unpleasant means that the situation should be further investigated. When the situation also is an actual *alarming situation* for patient Bob, it is called a *fault*. When a fault is detected, certain action(s) can/should be taken by the system to try to solve the fault. Whether or not a symptom implies a fault and thus requires action, depends on information regarding the actual context. For this use case, this is only true if the patient who is accommodated in the room where the threshold is crossed, is sensitive to the measured property, e.g., light intensity or sound.

Once a fault is detected, the system will try to solve it by taking one or more actions. An action can be static, or it can depend on other data. For this use case, the action taken depends on the presence of a nurse in the patient room at the time of the fault detection. When a nurse, who is responsible for that patient, is present in the room, the fault is considered less severe. In such a situation, it is likely to assume that the nurse knows how to treat the patient and that precautions have been taken to shield the patient from direct light and sound exposure. However, it might be useful to warn the nurse of the exceeded threshold by means of a message on an information display or on a mobile device. When no nurse is present in the room, the fault is much more severe. Actions should be taken to resolve the fault: a nurse should be called by the system to go on site and check the situation in the room. Awaiting the arrival of the nurse, local action can already be taken. For example, in case the fault is caused by a high light intensity observation, the light level can already be automatically reduced to a more acceptable level, e.g., by dimming the lights or closing the window blindings. Again, a warning can be displayed on an information display to make people in the room aware of what is happening.

### 4.2. Architectural Use Case Set-Up

**Figure 3:**
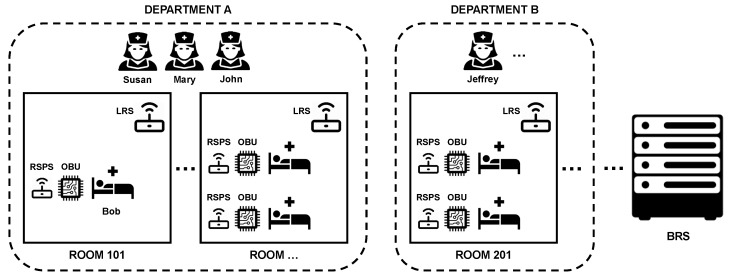
Architectural set-up for the Proof-of-Concept use case. Each hospital room contains one Local Reasoning Service (LRS), and one Observation Unit (OBU) and RSP Service (RSPS) per patient. There is only one Back-end Reasoning Service (BRS) in the hospital. Patient Bob is accommodated in room 101 of department A, which is supervised by nurses Susan, Mary and John.

To implement the use case, the architecture presented in Section 3 is used. An overview of the architectural set-up for this use case is shown in Figure 3.

To map the architecture, a few assumptions about the hospital and its rooms are made. The hospital consists of multiple departments. On each department, multiple nurses are working. In each department, several hospital rooms are located, both single-person and multi-person rooms. In each room, there exists exactly one OBU and RSPS per bed, i.e., if accommodated, per patient. There is one LRS per room, independent of the amount of patients. Over the full hospital, only one BRS exists. For this implementation, such a simple set-up is considered. However, in a real-life set-up, there will typically be more than one BRS component in the system, e.g., an extra BRS per department, as indicated in Figure 2.

In each room, the OBU consists of a BLE sensor, and multiple environmental and/or body sensors. For the concussion diagnosis and corresponding sensitiveness to light intensity and sound, a light and sound sensor are sufficient. Of course, in a real-life use case, the available medical domain knowledge will consist of multiple diagnoses. Accordingly, the OBU will then also consist of potentially other sensors. For the system to work correctly, accuracy of the sensors is required. For example, the range of the BLE beacon should be correctly configured, such that it does not detect BLE devices that are nearby, but in another room.

### 4.3. Continuous Care Ontology

A continuous care ontology has been designed [53] to describe existing medical domain knowledge, to enable the semantic annotation of the sensor observations, and to allow modeling all available context information. For PoC purposes, a new ontology has been designed for this. However, to link it with existing medical ontologies, a mapping to the SNOMED ontology can be added to the ontology.

The starting point for this ontology was the ACCIO ontology [54]. This is an OWL 2 DL ontology designed to represent different aspects of patient care in continuous care settings [55]. It links to other existing ontologies, such as the SSN [9] and SAREF [56] ontologies. For this use case, the ACCIO ontology allows to represent hospital departments, rooms, sensors, BLE bracelets, observations, nurses, patients, actions, nurse calls, etc. These concepts can be represented, as well as the relations between them. Moreover, the ontology contains some concepts for this use case that allow the inference of certain situations and hence easier query writing. An example is NursePresentObservation, which is defined as a BLE tag observation of a bracelet owned by a nurse:


NursePresentObservation ≡
    (hasResult some (
        observedDevice some (
            BLEBracelet and (deviceOwnedBy some (
                        Person and (hasRole some StaffMemberRole))))))
    and (madeBySensor some BLEBeacon).
        

The existing ACCIO ontology has been further extended for this work with some key concepts and relations specific for the current use case. This extension is the CareRoomMonitoring.owl ontology. Here, all possible medical symptoms, diagnoses, symptoms and faults are defined. For this use case, the Concussion class is defined as being equivalent to a Diagnosis that has two medical symptoms, being light sensitiveness and sound sensitiveness:


Concussion ⊑ Diagnosis
              and (hasMedicalSymptom some SensitiveToLight)
              and (hasMedicalSymptom some SensitiveToSound).
        

Moreover, the SoundAboveThresholdFault class is defined as:


SoundAboveThresholdFault ≡
  (hasSymptom some SoundAboveThresholdSymptom)
  and (madeBySensor some (
      isSubsystemOf some (
          hasLocation some (
              isLocationOf some (
                 (hasDiagnosis some (hasMedicalSymptom some SensitiveToSound))
                   and (hasRole some PatientRole)))))).
        

Two conditions need to be fulfilled for an Observation individual to also be a SoundAboveThresholdFault individual. First, it needs to have a SoundAboveThresholdSymptom, indicating the possibly unpleasant situation. Second, the observation needs to be made by a sensor system situated at the same location as a sound sensitive patient, i.e., a patient with a diagnosis that implies sound sensitiveness. If that is also the case, the possibly unpleasant situation is alarming. For this use case, the definition of LightIntensityAboveThresholdFault is completely similar.

The approach of using medical symptoms, diagnoses, symptoms and faults allows complete separation of diagnosis registration and fault detection. For an observation to be a fault, the exact diagnosis of the patient located at the corresponding room is unimportant; only the medical symptom, e.g., sensitiveness to light or sound, needs to be known. Vice versa, a patient’s sensitiveness to light and sound, or any other medical symptom, does not need to be explicitly registered in the system; registering the diagnosis is sufficient. Because the diagnosis is already defined in the system according to medical domain knowledge, its corresponding medical symptoms are implicitly known.

**Figure 4:**
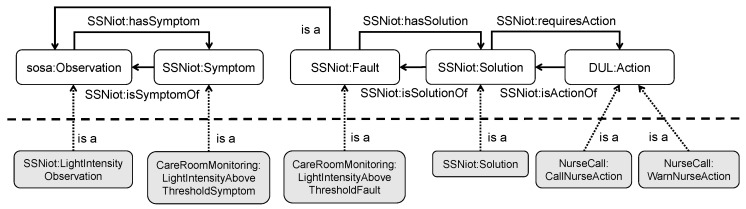
Overview of the most important ontology patterns and related classes of the Proof-of-Concept use case. The example for light intensity is given; the classes and patterns for sound are similar.

By design, the ACCIO ontology contains different patterns to represent the logic related to this use case. For example, a Fault is an Observation that needs a Solution through hasSolution, and a Solution requires an Action via requiresAction. Each such Action has exactly one Status via hasStatus, indicating the status in the life cycle of the action. To this end, a general overview of the most important ontology patterns and classes is presented in Figure 4.

### 4.4. Data Sources

As can be seen in Figure 1, each RSPS, LRS and BRS component of the system works with two data sources: the knowledge base and streaming data. Both are use case specific.

#### 4.4.1. Knowledge Base

For this use case, the knowledge base consists of the continuous care ontology, described in Section 4.3, and available context data. This information can be managed in a centralized database system, which can be an RDF triple store, or a database system that supports OBDA [52]. Examples of OBDA systems are Ontop [57] and Stardog [58]. By configuration, only the relevant parts of the knowledge base are available on each LRS and BRS. On the BRS, the full knowledge base is available.

Context data can be considered as static data: although it can change over time, the number of updates is low compared to the number of times a query evaluation is performed on the data before it changes. For this PoC, the context data includes information about the hospital layout, patients and nurses, the OBU and connected sensors, and BLE wearables. Changes to this context data are less frequent but do occur. For example, a newly diagnosed person is being accommodated in a room, or a new nurse starts working at a department. In these cases, the knowledge base of each relevant component needs to be updated. This is coordinated from the central knowledge base at the BRS.

On each RSPS and LRS component, only the information about the current department and room is known. For the nurses, context data related to all hospital nurses of the own department is available on each RSPS and LRS. On each RSPS, only the patient information of its associated patient is available. Similarly, patient data of all patients in the room is present in the knowledge base of each LRS. Consequently, on the RSPS and LRS of Bob’s room, four persons are defined: patient Bob, and three nurses, Susan, Mary and John. Bob is lying in room 101 of department A. According to the modeled diagnosis, he is suffering from a concussion. For both light intensity and sound, Bob’s threshold values are modeled. Moreover, each person is assigned a BLE bracelet.

The OBU in room 101 is uniquely identifiable by its MAC address. Three sensors are connected to the OBU: a sound sensor, a light sensor and a BLE beacon. Each sensor has a unique identifier composed of the MAC address of the OBU and a sensor ID which is unique on a single OBU.

Moreover, a threshold value is modeled for the light and sound sensor. These thresholds are identical to the corresponding threshold values of Bob for exposure to light intensity and sound. Directly linking these thresholds to the sensors themselves is crucial for the filtering at the RSP engine, as will be explained in Section 5.2. The process of mapping the thresholds of a person, related to a received medical diagnosis, to thresholds of the sensors of the patient’s OBU, needs to happen when a (new) diagnosis is made. This is achieved by running an appropriate query, inserting the sensor thresholds into the different knowledge bases.

Similarly to the patient data, only the associated OBU data is available in the knowledge base of an RSPS. On each LRS, all OBU data of the associated room is known.

#### 4.4.2. Streaming Data

For this use case, the streaming data is semantically annotated and pushed to different streams by an OBU. The semantic annotation is an important task. In this mapping process, the observations are modeled according to the continuous care ontology. Listing 1 shows the template of an RDF observation message for a sound observation: (A) denotes the sensor identifier, (B) the observation timestamp expressed in milliseconds, (C) the observation timestamp in xsd:dateTime format, (D) the observed value, and (E) the corresponding unit. The name of each observation is unique due to the observation identifier (A)-(B). For a light intensity or BLE tag observation, the template is similar. In case of a BLE tag observation, the result contains the ID of the observed BLE device.

**Listing 1.** Template of a semantically annotated sound sensor observation.
obs:Observation_(A)-(B) rdf:type sosa:Observation ;

  General:hasId [ General:hasID "(A)-(B)"^^xsd:string ] ;

  sosa:observedProperty [ rdf:type SSNiot:Sound ] ;

  sosa:madeBySensor [ General:hasId [ General:hasID "(A)"^^xsd:string ] ] ;

  sosa:resultTime "(C)"^^xsd:dateTime ;

  sosa:hasResult [ rdf:type SSNiot:QuantityObservationValue ;

                   DUL:hasDataValue "(D)"^^xsd:float ;

                   SSNiot:hasUnit "(E)"^^xsd:string ] .


## 5. PoC Component Implementation

**Figure 5:**
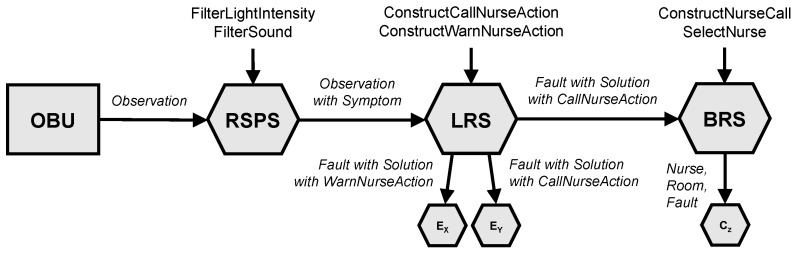
Overview of the components of the Proof-of-Concept. The inputs and outputs of each main component are shown in italic, as well as the queries executed on each RSP Service (RSPS), Local Reasoning Service (LRS) and Back-end Reasoning Service (BRS). The additional components E${}_{X}$ and E${}_{Y}$ represent the components taking local action. C${}_{Z}$ represents the component taking care of the actual nurse call. Feedback loops are omitted from the overview.

This section will go into detail on the implementation of the PoC, introduced in Section 4, on each of the four main architecture components of the cascading reasoning framework presented in Section 3. Figure 5 gives an overview of the main components of the PoC. It shows the inputs and outputs of each component, which are all events containing RDF triples, as well as the queries that are being executed on each RSPS, LRS and BRS component.

### 5.1. OBU

In every hospital room, one OBU per patient is present to monitor the environment. For this PoC, the single-person room of patient Bob is considered, where one OBU is installed. As explained in Section 4.4.1, this OBU consists of three sensors: a light sensor, a sound sensor and a BLE beacon. In a realistic system, the light and sound sensors can be part of a sensor board, such as a GrovePi+. As a BLE beacon is a different type of sensor, it is not part of this board. Therefore, for the implementation of this PoC, the OBU pushes the sensor observations in two separate RDF data streams: one stream (http://rspc1.intec.ugent.be/grove) containing the sensor board observations, and one stream (http://rspc1.intec.ugent.be/ble) containing the BLE tag observations. Recall that the architecture is push-based, i.e., each observation made by the sensors is immediately pushed on these streams.

### 5.2. RSPS

As explained in Section 3, the RSPS is situated locally, and consists of an RSP engine. In the given use case, locally means within the hospital room. In concrete, there is one RSPS component per OBU, i.e., per patient. Therefore, for this PoC, one RSPS component is deployed in Bob’s room.

For the RSPS, the used RSP engine is C-SPARQL [21], because of its support for static context data [20]. Both input and output of the RSP engine are RDF streams. It is used together with the RSP Service Interface for C-SPARQL [59], which offers a simple RESTful interface to interact with C-SPARQL. In terms of reasoning capabilities, C-SPARQL incorporates the possibility to perform RDFS reasoning. However, reasoning is time-consuming and may take too much time when fast reevaluation of the continuous queries is required. Therefore, for this use case, no reasoning is performed by C-SPARQL.

In the cascading reasoning approach, RSP engines are used to filter the change frequency of the data streams. Only interesting information is retained. Therefore, appropriate continuous queries are constructed that intelligently aggregate and filter the data streams for the use case at hand.

For this use case, two similar C-SPARQL queries are running on the RSPS component: FilterSound and FilterLightIntensity. As explained before, a window needs to be placed on top of the continuous data streams. For these queries, a logical window is used, which is a window extracting all triples occurring during a certain time interval. In concrete, both queries are executed every 5 s, on a logical sliding window containing all light, sound and BLE tag observations of the previous 6 s. The window size of 6 s is chosen as such to avoid situations where certain observations fall between two windows. Theoretically, this should not be possible when the window size and sliding step are both equal, but, in practice, a real implementation of the system may exhibit a lag of a few milliseconds between two query executions. The triples constructed by the query are sent as RDF messages to the event stream of the LRS. Listing 2 shows the FilterSound query, which is discussed in the next paragraphs. The FilterLightIntensity query and its motivation are completely similar.

Lines 14–16 of the FilterSound query define its inputs: the stream with sensor board observations, the stream with BLE tag observations, and the context data available in the local knowledge base.

The WHERE clause of the query consists of two large parts (lines 18–44 and lines 46–59). Considering the first part, its first section (lines 19–25) extracts any sound sensor observation in the input window. The second section (lines 27–41) contains an optional pattern that looks at BLE tag observations in the window corresponding to the BLE bracelet of a nurse. Note that this pattern only matches BLE tag observations of nurses that are working on the department the hospital room belongs to. This is because context information about the BLE bracelets of other nurses is not available in the local knowledge base. The rationale for this is that it is assumed that nurses of other departments know too little about the patients of the current department. Hence, their presence in the room should not affect the outcome of the query.

**Listing 2.**FilterSound query running on the RSP Service (RSPS) component.
1
CONSTRUCT {
2
    _:sym rdf:type CareRoomMonitoring:SoundAboveThresholdSymptom ;
3
          General:hasId [ General:hasID ?o_id ] .
4
    ?m_o SSNiot:hasSymptom _:sym ; rdf:type sosa:Observation ;
5
         General:hasId [ General:hasID ?o_id ] ; sosa:hasResult ?m_r .
6
    ?m_r DUL:hasDataValue ?m_v ; SSNiot:hasUnit ?u .
7
    ?m_o sosa:resultTime ?m_t ; sosa:madeBySensor ?s .
8
 
9
    ?ble_ob1 rdf:type sosa:Observation ; sosa:madeBySensor ?ble_s1 ;
10
             sosa:resultTime ?ble_ob1_time ; sosa:hasResult ?ble_r1 .
11
    ?ble_r1 rdf:type SSNiot:TagObservationValue ;
12
            SAREFiot:observedDevice ?ble_b1_id .
13
}
14
FROM STREAM <http://rspc1.intec.ugent.be/grove> [RANGE 6s STEP 5s]
15
FROM STREAM <http://rspc1.intec.ugent.be/ble> [RANGE 6s STEP 5s]
16
FROM <http://localhost:8181/context.ttl>
17
WHERE {
18
    {
19
        ?m_o rdf:type sosa:Observation ; sosa:hasResult ?m_r ;
20
             sosa:madeBySensor [ General:hasId [ General:hasID ?s_id ] ] ;
21
             General:hasId ?o_id_ent ; sosa:resultTime ?m_t .
22
        ?o_id_ent General:hasID ?o_id . ?m_r DUL:hasDataValue ?m_v .
23
        OPTIONAL { ?m_r SSNiot:hasUnit ?u } .
24
        ?s rdf:type SSNiot:LightSensor; General:hasId [ General:hasID ?s_id ] ;
25
           SSNiot:hasThreshold [ DUL:hasDataValue ?th ] .
26
 
27
        OPTIONAL {
28
            ?ble_ob1 rdf:type sosa:Observation ;
29
                     sosa:madeBySensor [ General:hasId [
30
                         General:hasID ?ble_s1_id ] ] ;
31
                     sosa:resultTime ?ble_ob1_time ; sosa:hasResult ?ble_r1 .
32
            ?ble_r1 rdf:type SSNiot:TagObservationValue ;
33
                    SAREFiot:observedDevice [ General:hasId [
34
                        General:hasID ?ble_b1_id ] ] .
35
            ?ble_s1 rdf:type SSNiot:BLEBeacon ;
36
                    General:hasId [ General:hasID ?ble_s1_id ] .
37
            ?ble_b1 rdf:type SAREFiot:BLEBracelet ;
38
                    General:hasId [ General:hasID ?ble_b1_id ] .
39
            ?p1 rdf:type DUL:Person ; SAREFiot:ownsDevice ?ble_b1 ;
40
              DUL:hasRole [ rdf:type RoleCompetenceAccio:StaffMemberRole ]
41
        }
42
 
43
        FILTER (xsd:float(?m_v) > xsd:float(?th))
44
    }
45
 
46
    FILTER (EXISTS {
47
        ?ble_ob2 rdf:type sosa:Observation ; sosa:hasResult ?ble_r2 ;
48
                 sosa:madeBySensor [ General:hasId [
49
                     General:hasID ?ble_s2_id ] ] .
50
        ?ble_r2 rdf:type SSNiot:TagObservationValue ;
51
                SAREFiot:observedDevice [ General:hasId [
52
                    General:hasID ?ble_b2_id ] ] .
53
        ?ble_s2 rdf:type SSNiot:BLEBeacon ;
54
                General:hasId [ General:hasID ?ble_s2_id ] .
55
        ?ble_b2 rdf:type SAREFiot:BLEBracelet ;
56
                General:hasId [ General:hasID ?ble_b2_id ] .
57
        ?p2 rdf:type DUL:Person ; SAREFiot:ownsDevice ?ble_b2 ;
58
            DUL:hasRole [ rdf:type RoleCompetenceAccio:PatientRole ] .
59
    })
60
}
61
ORDER BY DESC(?m_t)
62
LIMIT 1



Line 43 of the query contains an important filter. It checks for each sound observation whether the observed sound value is higher than the threshold of the sound sensor that measured the value. As explained in Section 4.4.1, this sensor threshold exactly corresponds to the patient’s sound exposure threshold defined in the patient’s EHR. Only if this threshold is crossed, the observation is retained, as this might imply a possibly unpleasant situation, i.e., a symptom.

The second part of the WHERE clause consists of a second filter clause (lines 46–59). This filter will ensure that the WHERE clause will only yield a result pattern, if there currently is a BLE tag observation present in the input window, which corresponds to a BLE bracelet of a patient. If this is not the case, no relevant patient is currently present in the hospital room. This means that the crossed sound threshold is not a problem. Note that, if the presence of a patient is detected, this automatically is the patient lying in the bed corresponding to the RSPS. This is again a consequence of the fact that the local knowledge base of the RSPS only contains patient information about its corresponding bed.

Lines 61–62 of the query sort the results of the WHERE clause according to the observation timestamp in descending order, and only retain the first result. In this way, if there are multiple sound observations above the sensor threshold, only the most recently observed sound value is retained in the query result.

If the query yields a result, new triple patterns are constructed by the CONSTRUCT part (lines 2–12). The high sound observation, which crossed its sensor’s threshold, will be given a newly created SoundAboveThresholdSymptom. Other information on the particular observation is copied as well, together with any BLE tag observation of a nurse’s bracelet, detected through the OPTIONAL clause.

### 5.3. LRS

The LRS is situated in the edge of the network, as discussed in Section 3. In this use case, this again corresponds to the hospital room. Therefore, in this PoC, there is one LRS per hospital room.

#### 5.3.1. Reasoning Service

To implement the LRS, and also the BRS addressed in Section 5.4, a reasoning service component is implemented. In this reasoning service, the OWL 2 RL reasoner RDFox [14] is used. It was chosen over other, more expressive reasoners such as HermiT because of its highly efficient reasoning [14].

The continuous care ontology presented in Section 4.3 is an OWL 2 DL ontology, whereas RDFox is an OWL 2 RL reasoner. The OWL 2 RL profile is designed to implement reasoning systems using rule-based reasoning engines [60]. Therefore, certain restrictions are present. One restriction is that an OWL 2 RL reasoner cannot infer the existence of individuals that are not explicitly present in the knowledge base. For example, according to the OWL 2 DL definition of Concussion presented in Section 4.3, for each Concussion individual p, an OWL 2 DL reasoner will create new triples p rdf:type Diagnosis, p hasMedicalSymptom [ rdf:type SensitiveToLight ] and p hasMedicalSymptom [ rdf:type SensitiveToSound ]. An OWL 2 RL reasoner cannot infer the second and third triple, which forms a problem, as their existence is used in the SoundAboveThresholdFault definition. Therefore, such triples are explicitly added to the ontology for a single Concussion individual, which is then used to model the diagnosis of a person.

By configuration, the reasoning service has a number of predefined event-based processing SPARQL queries. SELECT, CONSTRUCT and UPDATE queries are supported. Importantly, these queries can be ordered in the component’s configuration file. This is required because they can depend on each other: an UPDATE query may for example work on new triples constructed by a CONSTRUCT query.

For a CONSTRUCT and SELECT query, one or more observers can be defined to which the resulting triples need to be sent. These observers are endpoints, such as streams. For example, in the LRS, the triples constructed by a CONSTRUCT query can be sent to the event stream of the BRS. Variable bindings outputted by SELECT queries can also be sent to external components for further processing. For a CONSTRUCT query, it can also be defined whether the constructed triples should be added to the local triple store. UPDATE queries only update the triples in the local triple store. After every addition or removal of triples to/from the triple store, incremental OWL 2 RL reasoning is performed. Incremental reasoning is a technique where the implicit assertions in the knowledge base are incrementally maintained, to avoid recomputing all assertions each time a new set of triples is added [14]. This technique is often employed by semantic query and reasoning engines to improve reasoning performance.

**Figure 6:**
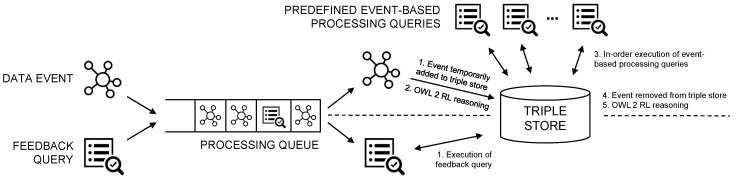
Overview of the functionality of the implemented reasoning service, used by the Local Reasoning Service (LRS) and Back-end Reasoning Service (BRS).

In Figure 6, an overview of the functionality of the reasoning service is given. The main running thread of the reasoning service component works with a single queue of incoming data events and feedback queries. This means that data events and feedback queries can be sent to the system, where they are added to the queue and sequentially processed in order of arrival.

In this use case, a data event is an RDF message that is arriving from an RSPS, LRS or BRS component. When the event is removed from the queue by the main running thread, i.e., when the processing starts, the event triples are temporarily added to the triple store. Next, incremental OWL 2 RL reasoning is performed, generating all inferred triples. Each predefined query is then sequentially executed in the defined order. When finished, the event triples are again removed from the triple store, after which a final incremental reasoning step is executed.

Besides the event-based processing queries that are executed on every event, in some cases, it might be interesting to execute a specific query once on the triples of the triple store, e.g., when the status of an action needs to be updated. Such feedback queries can also be sent to the system. When a feedback query is processed by the reasoning service, it is simply executed on the local triple store. Because these feedback queries are straightforward for this use case, they are not further discussed.

#### 5.3.2. Event-Based Processing Queries

For this use case, incoming events at the LRS are RDF messages containing the triples constructed by the FilterSound (Listing 2) and FilterLightIntensity queries running on the RSPS. These events contain light and sound observations that are possibly unpleasant, i.e., that have a symptom. The following queries are sequentially run on the LRS when an event is being processed:

- ConstructCallNurseAction (Listing 3): This CONSTRUCT query looks for an instance of a DetectedFault (lines 12–14), i.e., it analyzes whether any fault is detected by the system. Recall that an observation is a fault if given conditions are fulfilled. For each fault, these conditions are integrated in the specification of the fault in the ontology. In this way, when the conditions are fulfilled, the fault has been inferred by the reasoner after the addition of the event triples.
  If a fault is detected—assuming the three filter clauses, addressed in the following paragraph, are passed—new triples are constructed (lines 2–8). A solution for the fault is created. Each solution requires a corresponding action, in this case a CallNurseAction. This action implies that a nurse should be called to go on site to check the room. A CallNurseAction individual has four statuses in its life cycle: New, when the action is created but not handled yet; Active, when a component has activated the action and is starting the nurse assignment process; Assigned, when a nurse has been assigned; and Finished, when the nurse has arrived at the room.
  Three FILTER NOT EXISTS patterns are added to the query’s WHERE clause. Only if they are passed, a solution is created. The first filter (line 17) ensures that no NursePresentObservation is present. Recall from Section 4.3 that this is a BLE tag observation that is associated with the BLE bracelet of a nurse. If this information is available in the event, this means that the RSPS has detected the presence of a nurse in the room. Obviously, no nurse should be called in this case; another action is then taken by the ConstructWarnNurseAction query. The second filter (lines 19–22) ensures that not more than one solution is created for a fault, i.e., an observation, with the same ID. The third filter (lines 24–32) clause avoids that a CallNurseAction is created when there is already another CallNurseAction for that room that is not finished yet. In that case, it makes no sense to call a nurse for the second time. Note that this only holds if the unfinished CallNurseAction corresponds to a fault of the same type. The rationale for this is that a new call to the same room, but, for another fault, gives new information to a nurse that he/she can for example take into account when deciding how urgent the call is. Note that the concept NewOrActiveOrAssignedAction is used for this filter. This concept is defined as a subclass of Action that has one of these three statuses.
  The triples constructed by the ConstructCallNurseAction are sent to the event stream of the BRS because this component will decide how to handle the CallNurseAction. To keep track of the status of the action, and to perform collect filtering in later executions of the query, the triples are also added to the local triple store.
     
- ConstructWarnNurseAction (Listing 4): This CONSTRUCT query is complementary to the ConstructCallNurseAction query in Listing 3. Hence, most parts are very similar.
  In the CONSTRUCT part of the query (lines 2–8), a WarnNurseAction is created instead of a CallNurseAction. In other words, a specific nurse p1 should be warned, instead of calling a nurse to the room. This nurse will be present in the room at the time of the threshold crossing.
  To only construct this particular solution when a nurse is present, the WHERE class of the query is extended with the pattern in lines 16–19. This extension ensures that a NursePresentObservation is present, and retrieves the associated nurse. The two filters of the query are similar to the second and third filter of the CallNurseAction query. The second filter (lines 27–36) ensures that no new WarnNurseAction is created for the present nurse if she is currently being warned already.
  The triples constructed by this query are not sent to the BRS because each WarnNurseAction is completely handled locally. However, the triples are again added to the local triple store.

**Listing 3.**ConstructCallNurseAction query running on the Local Reasoning Service (LRS) component.
1
CONSTRUCT {
2
    _:f rdf:type ?t1 ; General:hasId [ General:hasID ?id ] ;
3
        sosa:madeBySensor ?sen ;
4
        SSNiot:hasSolution [
5
            rdf:type SSNiot:Solution ;
6
            SSNiot:requiresAction [
7
                rdf:type NurseCall:CallNurseAction ;
8
                General:hasStatus TaskAccio:New ] ] .
9
}
10
WHERE {
11
    {
12
        ?f1 rdf:type ?t1 ; General:hasId ?idobj ; sosa:madeBySensor ?sen .
13
        ?idobj General:hasID ?id . ?sen SSNiot:isSubsystemOf ?sys .
14
        ?t1 rdfs:subClassOf SSNiot:DetectedFault .
15
    }
16
 
17
    FILTER NOT EXISTS { ?ble_ob1 rdf:type NurseCall:NursePresentObservation . }
18
 
19
    FILTER NOT EXISTS {
20
        ?f3 SSNiot:hasSolution ?s1 ; General:hasId ?f3_idobj .
21
        ?f3_idobj General:hasID ?id .
22
    }
23
 
24
    FILTER NOT EXISTS {
25
        {
26
            ?f2 rdf:type ?t1 ; SSNiot:hasSolution ?s2 .
27
            ?s2 SSNiot:requiresAction ?a2 .
28
            ?a2 rdf:type NurseCall:CallNurseAction,
29
                         TaskAccio:NewOrActiveOrAssignedAction .
30
        }
31
        FILTER (?f1 != ?f2)
32
    }
33
}



**Listing 4.**ConstructWarnNurseAction query running on the Local Reasoning Service (LRS) component.
1
CONSTRUCT {
2
    _:f rdf:type ?t1 ; General:hasId [ General:hasID ?id ] ;
3
        sosa:madeBySensor ?sen ;
4
        SSNiot:hasSolution [
5
            rdf:type SSNiot:Solution ;
6
            SSNiot:requiresAction [
7
                rdf:type NurseCall:WarnNurseAction ;
8
                DUL:hasContext ?p1 ; General:hasStatus TaskAccio:New ] ] .
9
}
10
WHERE {
11
    {
12
        ?f1 rdf:type ?t1 ; General:hasId ?idobj ; sosa:madeBySensor ?sen .
13
        ?idobj General:hasID ?id . ?sen SSNiot:isSubsystemOf ?sys .
14
        ?t1 rdfs:subClassOf SSNiot:DetectedFault .
15
 
16
        ?ble_ob1 rdf:type NurseCall:NursePresentObservation ;
17
                 sosa:hasResult ?ble_r1 .
18
        ?ble_r1 SAREFiot:observedDevice ?ble_b1 .
19
        ?p1 SAREFiot:ownsDevice ?ble_b1 .
20
    }
21
 
22
    FILTER NOT EXISTS {
23
        ?f3 SSNiot:hasSolution ?s1 ; General:hasId ?f3_idobj .
24
        ?f3_idobj General:hasID ?id .
25
    }
26
 
27
    FILTER NOT EXISTS {
28
        {
29
            ?f2 rdf:type ?t1 ; SSNiot:hasSolution ?s2 .
30
            ?s2 SSNiot:requiresAction ?a2 .
31
            ?a2 rdf:type NurseCall:WarnNurseAction,
32
                         TaskAccio:NewOrActiveOrAssignedAction ;
33
                DUL:hasContext ?p1
34
        }
35
        FILTER (?f1 != ?f2)
36
    }
37
}



#### 5.3.3. Taking Local Action

The ConstructWarnNurseAction query running on the LRS creates a WarnNurseAction when a nurse is in the room and should be warned of a fault. To do so, an external component should be involved that is capable of communicating with the wearables, i.e., that can transfer the triples constructed by the query into a real notification on the nurse’s wearable. This component should therefore be registered as observer of the ConstructWarnNurseAction query. It is also the task of this component to correctly update the status of the WarnNurseAction individual. This can be done by sending appropriate UPDATE queries to the LRS to delete and insert the correct triples in the local knowledge base. In Figure 5, this component is shown as component E${}_{X}$.

An example of a system that could be used for this additional component is DYAMAND [61]. This is a framework that acts as a middleware layer between applications and discoverable or controllable devices. It aims to provide the necessary tools to overcome the interoperability gaps that exist between devices from the same and other domains.

For the ConstructCallNurseAction query, the situation is different compared to the ConstructWarnNurseAction query. Constructed CallNurseAction individuals are sent to the BRS, where they are handled further. This means that the BRS, or another component interacting with the BRS, is responsible for updating the status of each CallNurseAction in the local knowledge base. Nevertheless, this does not mean that the LRS cannot take local action. Again, a component such as DYAMAND can be registered as observer to the ConstructCallNurseAction query. Based on the fault associated to an incoming CallNurseAction, it can already take some first local action. For example, in case of a LightIntensityAboveThresholdFault, the component can check whether the lights are switched off and the window blindings are closed. If not, this can then automatically be done. In Figure 5, this component is shown as component E${}_{Y}$.

### 5.4. BRS

As addressed in Section 3, the BRS is running in the cloud of the network. Only one BRS is available for the entire hospital. The BRS has access to the full central knowledge base.

In the BRS, an ontology-based nurse call system (oNCS), similar to the one presented by Ongenae et al. [62], should be deployed. This includes complex probabilistic reasoning algorithms that determine the priority of a nurse call, based on the risk factors of the patient.

For this PoC, an incoming event on the BRS is the output of the ConstructCallNurseAction query. The following queries are sequentially run each time an event is processed:

- ConstructNurseCall (Listing 5): This CONSTRUCT query handles any CallNurseAction with status New that has been created as solution for a detected fault. It transforms the scheduled action into an actual nurse call. The query extracts the sensor system that caused the fault, which is set to have made the call. The reason for the call is the fault that the solution and action were created for. The triples constructed by this query are only added to the local triple store.
- SelectNurse (not listed): This query represents the complex nurse assignment process of the oNCS. For every nurse call constructed by the previous query, it should assign a nurse to the call. Again, a separate component—observing the results of this query, and using a system such as DYAMAND—can be used to actually show the call to the nurse on his/her wearable. Important information for the nurse includes the room, patient and reason for the call, i.e., the fault. In Figure 5, this additional component is shown as component C${}_{Z}$.

Note that the BRS is also responsible for keeping the status of each CallNurseAction and ContextCall at the corresponding LRS up to date.

**Listing 5.**ConstructNurseCall query running on the Back-end Reasoning Service (BRS) component.

CONSTRUCT {

    _:c rdf:type NurseCall:ContextCall ;

        NurseCall:callMadeBy ?sys ; General:hasStatus TaskAccio:New ;

        General:hasId [ General:hasID ?f_id ] ;

        TaskAccio:hasReason [ rdf:type ?t ] .

}

WHERE {

    ?f rdf:type ?t ; General:hasId ?f_idobj ;

       SSNiot:hasSolution ?sol ; sosa:madeBySensor ?s .

    ?t rdfs:subClassOf SSNiot:DetectedFault . ?s SSNiot:isSubsystemOf ?sys .

    ?f_idobj General:hasID ?f_id . ?sol SSNiot:requiresAction ?act .

    ?act rdf:type NurseCall:CallNurseAction ; General:hasStatus TaskAccio:New .

}



### 5.5. Platform Configuration

To adopt the implementation of the cascading reasoning framework for a specific use case, a number of things should be configured, like it has been described for the PoC use case. First, the network of components should be defined. The domain ontologies and context information should be described, and it should be configured which parts are relevant for which components. Moreover, the OBU should be configured to map its observations to RDF messages. Furthermore, for each RSPS component, the continuous queries should be defined. Similarly, appropriate event-based processing queries should be configured for the LRS and BRS service. For each component, its inputs should be defined by selecting the queries of other components to observe, or the output stream of an OBU. Potential use case specific feedback loops and external components to perform any actions should additionally be added. By configuring the aforementioned things, the implementation of the framework can be applied to other use cases with minimal effort.

## 6. Evaluation Set-Up

To demonstrate the functionality and evaluate the performance of the presented cascading reasoning framework, evaluation scenarios have been executed on the PoC implementation presented in Section 4 and Section 5. Instead of using real sensors configured by the OBU, realistic scenarios have been created and simulated.

### 6.1. Evaluation Goals

The first goal of the evaluation is to verify the correct working of the system in multiple scenarios. In particular, it is interesting to look how the system behaves in three distinct cases: one case where no fault occurs, one case where a fault is handled locally, and one case where a fault is handled globally.

The second evaluation goal is to verify the global performance of the cascading reasoning platform. According to legal stipulations in some countries, a nurse should have arrived at the correct location at most five minutes after a fault has occurred. Given this condition, according to the manufacturer cooperating with the research group, i.e., Televic NV (Izegem, Belgium), each nurse call assignment should be completed within 5 s after the fault occurrence, i.e., after the observation that crossed the threshold. This leaves ample time for the nurse to move to the room after receiving the alert. Note that, by definition, the actual call of the assigned nurse, which needs to be performed by an external component, is not part of the assignment process. This process finishes when the decision on which nurse to call is made.

Related to the second goal, the third evaluation goal is to get insight into the component-level performance of the cascading reasoning platform. This includes looking at the processing time and latency on each component, network latency, etc.

### 6.2. Evaluation Scenarios

As indicated in the evaluation goals, three distinct scenarios are evaluated. Each scenario considers the use case and implementation of the PoC described in Section 4 and Section 5. For these scenarios, a few assumptions are made:

- The evaluation scenarios consider the architectural use case set-up presented in Figure 3. They only consider room 101 of department A, where patient Bob is accommodated. Bob is diagnosed with a concussion.
- The OBU in Bob’s room consists of exactly three sensors: a light sensor, a sound sensor, and a BLE beacon. The light sensor and sound sensor are producing one observation every second. The BLE sensor produces one or more observations every 5 s.
- All nurses of department A have a work shift comprising the whole period of the scenario.
- The domain knowledge in the knowledge base of each component consists of CareRoomMonitoring.owl and NurseCall.owl of the designed continuous care ontology. At the time of writing, the medical domain knowledge in CareRoomMonitoring.owl only contains one diagnosis, being concussion, and two medical symptoms, being sensitiveness to light and sound.
- The static context data in the local knowledge base of the RSPS and LRS exactly matches the context data described in Section 4.4.1. On the BRS, the knowledge base also contains similar context data about other rooms and patients. For this evaluation, it is assumed the hospital has two departments with 10 single-person rooms each. In each room, one OBU is present containing a light, sound and BLE sensor. Each room contains one diagnosed patient. On each department, three nurses are working during the current shift.
- On each component, the queries as given in Section 5 are running. To focus on the evaluation of the cascading platform, and not on the specific nurse call algorithm used, no full-fledged oNCS implementation is used on the BRS. Instead, a more simple version of the SelectNurse query is running, selecting one nurse of the department to assign to each nurse call.
- During the scenario runs, no background knowledge or context data updates are sent out from the BRS to the local knowledge bases.
- No real external components are actively present in the evaluation architecture. This is purely for evaluation purposes, as the goal is to look at the main components. As a consequence, no actual local actions are taken by real components. However, one mock-up external component exists, which is registered as an observer to the ConstructWarnNurseAction query on the LRS and the SelectNurse query on the BRS. In this way, the reasoning services send outgoing events (query results) to this component, allowing for calculating the latency of the components.
- Action statuses are also not automatically updated by feedback loops. Instead, the evaluation scripts ensure that, after each scenario run, the components are restarted, resetting the knowledge bases of all components to the pre-scenario state.
- During the scenario runs, all nodes are fully available. No loss of connectivity to the cloud occurs.

For all three scenarios, the preconditions are the same. Patient Bob is the only person present in hospital room 101. As Bob is recovering from a concussion, measures have been taken to protect him from direct exposure to light and sound. Therefore, the lights are dimmed, the window blindings are closed, and the television in the room is switched off. The light intensity is stable at 125 lumen, and the sound is stable at 10 decibels.

**Baseline scenario.** During this scenario, the light intensity and sound values remain stable at 125 lumen and 10 decibels, respectively. No threshold is crossed, so that no symptom is created or fault is inferred. Hence, no nurse should be warned or called.

In both scenarios 1 and 2, assume Bob’s wife enters the room after 30 s. Upon entering the room, she turns on the lights. As a result of turning on the lights, observed light intensity values rise up to 400 lumen.

**Scenario 1.** Assume nurse Susan is present in Bob’s room at that moment. Normally, Susan should know Bob’s diagnosis, and hence directly tell Bob’s wife to turn off the lights again. However, assume the system will notify Susan on her bracelet that Bob is sensitive to light and that there is currently too much light intensity exposure for him. This happens at the LRS through an extra component, as explained in Section 5.3. As a consequence, after 10 s, the lights are turned off again, and the observed light intensity values decrease to 125 lumen. The BRS is not involved in this scenario, as no nurse should be called. After 20 more seconds, Susan leaves the room, and the scenario ends.

**Scenario 2.** Assume no nurse is present in Bob’s room when his wife enters and turns on the lights. Hence, a nurse call will be initiated by the system, involving the RSPS, LRS and BRS components. In the BRS, nurse Susan is selected (**scenario 2a**). Thirty seconds later, Susan arrives at the room, explains to Bob’s wife that she should not turn on the lights, and turns them off. Hence, five seconds after Susan’s arrival, the observed light intensity values decrease to 125 lumen. Within this short time frame, Susan will receive another warning on her bracelet, triggered by the LRS (**scenario 2b**). Again, Susan stays for 20 s in the room. Afterwards, she leaves the room, and the scenario ends.

In Table 3, an overview is given of the observed values of the light sensor and the BLE sensor, for both scenarios 1 and 2.

Each scenario has been simulated 50 times. For each scenario, Gaussian noise with a variance of 1 has been added to the light intensity and sound observations. For all communication between components, the HTTP protocol is used. Hence, pushing an observation or query result on a component’s stream means sending an HTTP POST request to the stream endpoint containing the data in the request body. The following metrics are calculated:

- **Network latency** of an event *X* from A to B: This is the time between the outgoing event *X* at component A and the incoming event *X* at component B.
- **RSPS processing time** and **RSPS latency**: Processing on the RSPS is not event-based, but window-based, i.e., time-based for the FilterSound and FilterLightIntensity queries. Say an observation event *X* arrives at the RSPS at time $t_{X}$. If this observation leads to a symptom, one of both queries will construct an output containing a symptom for observation *X*. Say $t_{Y}$ is the time at which this RSPS query output is sent to its observers. The RSPS processing time for that observation is then defined as $t_{Y}-t_{X}$. By definition, the RSPS latency equals the RSPS processing time. Note that, by definition, the RSPS processing time is not defined for observations that do not lead to a symptom.
- **LRS processing time**, **LRS latency** and **LRS queuing time**: Processing on the LRS is event-based. When an event arrives at $t_{in}$, the event is put in the queue. After the queuing time, the processing of the event starts at $t_{start}$. The event is added to the knowledge base, and queries are executed. An event can only lead to either a CallNurseAction or WarnNurseAction, but not both, i.e., only one LRS query can construct a result. If this is the case, say $t_{out}$ is the time at which this outgoing event is sent to its observers. After all queries are executed, the event is removed from the knowledge base and processing ends at $t_{end}$. Given these definitions, the LRS processing time is defined as $t_{end}-t_{start}$; the LRS latency, if an outgoing event is sent, as $t_{out}-t_{in}$; and the LRS queuing time as $t_{start}-t_{in}$.
- **BRS processing time**, **BRS latency** and **BRS queuing time**: Definitions are similar to the LRS case. The outgoing event corresponding to the BRS latency always contains the nurse that is assigned to the nurse call by the SelectNurse query.
- **Total system latency**: The total system latency is defined as the total time that an observation is in the system. For an observation that generates a WarnNurseAction, this is the time until the WarnNurseAction is created and can be sent to an external component. Hence, it is the sum of the OBU-RSPS network latency, RSPS latency, RSPS-LRS network latency and LRS latency. For an observation that generates a CallNurseAction, this is the time until a nurse is assigned on the BRS. Hence, the system latency is the sum of of the OBU-RSPS network latency, RSPS latency, RSPS-LRS network latency, LRS latency, LRS-BRS network latency and BRS latency.

### 6.3. Hardware Characteristics

To perform the evaluation, the architecture components in the evaluation set-up have been implemented as Docker containers on real hardware components. Hardware characteristics of the device running the OBU container are omitted because the observations are simulated. Hence, results do not depend on the performance of this component; the OBU just sends the observations to the RSPS streams. To host the RSPS, an Intel NUC, model D54250WYKH, is used. This device has a 1300 MHz dual-core Intel Core i5-4250U CPU (turbo frequency 2600 MHz) and 8 GB DDR3 1600 MHz RAM [63]. The edge component hosting the LRS also is an Intel NUC with the same model number and specifications. In the cloud, the BRS is hosted by a node on Virtual Wall 1 of the imec iLab.t testbeds Virtual Wall [64]. This node has a 2000 MHz dual-core AMD Opteron 2212 CPU and 4 GB DDR2 333 MHz RAM.

## 7. Evaluation Results

For each evaluation scenario, Table 4 shows the average amount of incoming RDF events on each component. These results are now described in detail for each scenario.

In the baseline scenario, on average 131.94 events have come in on the RSPS. Except for three runs, 132 events have arrived over the 60 s: 60 light intensity observations, 60 sound observations, and 12 BLE observations (1 every 5 s). No threshold is crossed, so no event is sent to the LRS. Hence, the LRS and BRS have no incoming events.

In scenario 1, there have been on average 143.86 incoming events on the RSPS. During a period of 10 s, the light intensity threshold is crossed, and the FilterLightIntensity query, executing every 5 s on a window with a size of 6 s, generates either two or three symptoms. These symptoms are sent to the LRS. There, only the first incoming event triggers the creation of a WarnNurseAction. This is handled locally, so no event is sent to the BRS.

In scenario 2, on average 179.68 events have arrived at the RSPS. Now, the light intensity threshold is crossed during a period of 35 s. Hence, seven or eight symptoms are generated by the FilterLightIntensity query. The first incoming event at the LRS generates a CallNurseAction, which is sent to the BRS. Hence, only one event arrives at the BRS. By the time the assigned nurse arrives at the room, the final one or two outgoing events at the RSPS also contain a nurse BLE observation, next to the symptom. Hence, at the LRS, a WarnNurseAction is constructed in addition. Again, by definition, this is handled locally, so no additional event is sent to the BRS.

**Figure 7:**
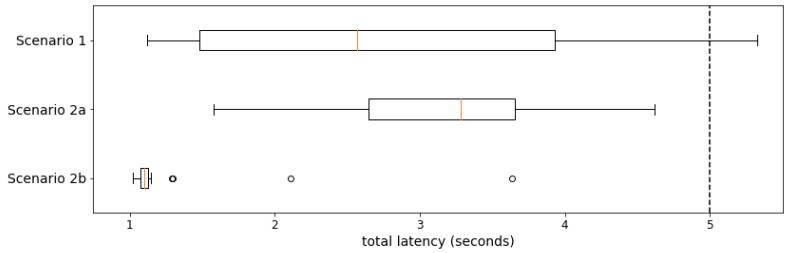
Boxplot showing the distribution, over all scenario runs, of the total system latency of three types of observations: the observation in scenario 1 causing a WarnNurseAction handled locally, the observation in scenario 2a leading to a CallNurseAction handled by the back-end, and another observation in scenario 2b that causes the creation of another WarnNurseAction. The vertical dashed line indicates the 5 s threshold, which is the targeted maximum system latency.

**Figure 8:**
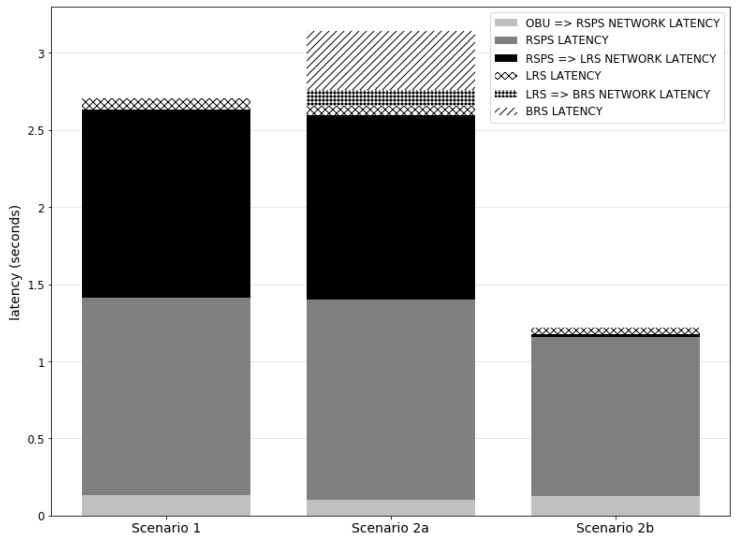
Bar plot showing the average total system latency of three types of observations, over all scenario runs: the observation in scenario 1 causing a WarnNurseAction handled locally, the observation in scenario 2a leading to a CallNurseAction handled by the back-end, and another observation in scenario 2b that causes the creation of another WarnNurseAction. For each situation, the different network and component latencies that sum up to the total system latency are indicated by the stacked bars.

Each observation that leads to the construction of an action on the LRS needs to be handled by the system. In scenario 1, one WarnNurseAction is generated, which is handled locally. In scenario 2, first a CallNurseAction is sent to the BRS (scenario 2a), and then a local WarnNurseAction is generated (scenario 2b). In Figure 7, a boxplot of the total system latency is shown for these three situations. Figure 8 shows the average total system latency for the three cases, split into the different component and network latencies.

For scenario 1, i.e., the situation with a WarnNurseAction, the total system latency is below the targeted maximum latency of 5000 ms in more than 90% of the runs. In concrete, it only rises up to 325 ms above 5000 ms in three runs. As observed in Figure 8, the average system latency is 2703 ms. The two largest parts of this system latency are the RSPS latency and the RSPS-LRS network latency. Fluctuations in these two latencies cause the large spread in system latency. The distribution of the RSPS latency has a positive skew, i.e., its right tail is longer. There is a significant amount of outliers on this side, i.e., runs with a higher RSPS latency. This is true for all three scenarios. The LRS processing time is on average 98 ms, which is slightly larger than the average LRS latency of 69 ms.

In scenario 2a, a CallNurseAction is handled by the BRS. The observation needs to pass through all the main architecture components, causing the largest average system latency of 3142 ms. Again, the RSPS latency and RSPS-LRS network latency make up the largest part. The BRS latency is on average 384 ms. However, the average BRS processing time is 28413 ms, indicating an additional processing time after the query executions of approximately 28 s on average. By definition, this large additional processing time does not cause any additional latency.

Scenario 2b involves an additional WarnNurseAction. Here, the average system latency is only 1215 ms. The average RSPS-LRS network latency is significantly smaller compared to the other two cases. The average RSPS latency is slightly smaller, and the other average latency values are comparable.

Finally, note that the queuing time on the LRS and BRS components is negligible for all runs of all scenarios.

## 8. Discussion

The results of the performance evaluation described in Section 7 allow to verify the evaluation goals explained in Section 6.1. As explained in the second evaluation goal, each nurse call assignment should be completed within 5 s after the fault occurrence, i.e., after the observation that crossed the threshold. Translating this to the evaluation results, this means that the system latency should be lower than 5 s. As can be observed in Figure 7, this was the case in all runs of scenario 2a for the CallNurseAction. However, one remark should be made concerning the simple SelectNurse query running on the BRS, instead of a complex oNCS. In fact, the time of a complex nurse call assignment algorithm should be added to the BRS latency. Considering such a very complex algorithm, such an assignment takes around 550 ms [65]. Adding this number, only three runs exceed the threshold of 5000 ms, and not by more than 250 ms. Ideally, the decision to warn a nurse is also taken within 5 s. Except for three runs in scenario 1, this requirement is also met.

A remark should however be made regarding the RSPS latency. This latency is directly dependent on the chosen query sliding step, i.e., the period between two executions of the query. This period defines the worst case scenario for the RSPS latency. In the case of the FilterLightIntensity query, which is similar to the FilterSound query in Listing 2, this sliding step is 5 s. The RSPS latency, equal to the RSPS processing time, is the time between the observation arrival and the start of the query execution, summed up with the duration of the query execution itself. If a threshold crossing observation arrives at the RSPS right after the start of the query execution, the first component of this sum can already be up to 5 s. Hence, to have more guarantees that the total system latency is always below 5 s, the frequency of the query execution should be increased. However, for this PoC, the FilterLightIntensity query creates a symptom for the most recent light intensity observation above the threshold. It is this observation that propagates through the system, and the associated observation time is used to calculate the system latency. When a threshold in light intensity is crossed, this will often be the case for multiple consecutive seconds, as is also the case in the evaluation scenarios. Hence, in most cases, when a threshold is crossed, this will be the case for the most recent observation in the window. As light intensity observations arrive at the RSPS every second, most RSPS processing times are around one second. In some cases, the most recent threshold crossing observation had arrived more than one second before the next query execution, explaining the RSPS processing times that are higher. In summary, the system latency is always below 5 s in the performed evaluation, but a few older observations in the window could already have been crossing the threshold.

Inspecting the breakdown of the total latency into component and network latencies in Figure 8, and associated numbers presented in Section 7 (evaluation goal 3), some other remarks can be made. First, the RSPS-LRS network latency is on average quite high in the case of the WarnNurseAction in scenario 1 and the CallNurseAction in scenario 2a. These actions always correspond to the first outgoing event of the scenario at the RSPS. As components were restarted after each scenario run in the evaluation, this was always the first outgoing event at runtime. As can be observed in the case of the WarnNurseAction in scenario 2b, the RSPS-LRS system latency is way lower on average for the following outgoing events. In real-life scenarios, components will not be restarted often. Hence, this network latency will be smaller on average. Second, the additional processing time after the query executions on the BRS is 28 s. This is caused by the removal of the data event and consecutive incremental reasoning step in RDFox. This large processing time does not affect the BRS latency, but is nevertheless not ideal. Hence, further research should be done on the reasoning service to decrease this value. Third, queuing time on the BRS is negligible in the current evaluation set-up. In real scenarios, other components may also send events to the BRS, causing a longer average and worst case waiting time. As the amount of events arriving at the BRS is, however, small in the average situation, the real impact may be limited. Fourth, as Table 4 indicates, the amount of incoming events on the components is not equal in each scenario run. In the three scenarios, 132, 144 and 180 events are generated by the OBU, respectively. Depending on the exact timing of the query executions, the last threshold crossing observation is present in either one or two query execution windows. This explains why, for scenario 1, the amount of incoming events of the LRS varies between 2 and 3. Similarly for scenario 2, the LRS receives either seven or eight incoming events. In each run, the amount of incoming events on the BRS is however the same.

The execution of the evaluation scenarios shows that a cascading reasoning system is well-suited for the described pervasive health use case. When no alarming situation occurs (baseline scenario), all events are filtered out by the RSPS. In this case, it is useless to contact the LRS or BRS. In the case where a potential alarming situation occurs, the RSPS just sends the event to the LRS, as the RSPS performs no reasoning. The LRS will process the event and reason on the data to infer whether or not it is a real alarming situation, i.e., a fault. If a fault is inferred and can be handled locally, it will be handled locally, and the BRS will also not be contacted (scenario 1). In this use case, this is when a nurse is present in the room. In that case, the local component knows perfectly what to do without any additional missing information: it should warn the nurse of the fault. The BRS is contacted (scenario 2) only if no nurse is present, as the LRS does not know by itself what nurses are available, where they currently are, what their current occupation is, etc. The BRS does know this information and is best-suited to decide on which nurse to call. In real-life situations, the BRS may require additional information to select and assign a nurse to a call. For example, every BLE observation of any nurse can be sent via the RSPS and LRS to the BRS, using additional simple queries. In this way, the BRS knows for each nurse when he/she is in a hospital room.

In its current implementation, the system performs two continuous checks for a concussion patient: it observes the light intensity and sound in the room. In real-life situations, the system may also need to monitor other alarming situations, e.g., when a patient has fallen on the ground. Adding these additional checks is easily possible by incorporating the required sensors, extending the domain ontology accordingly, providing the appropriate context information, and registering the corresponding queries on the different components. In some situations, priorities concerning the alarming situations and corresponding actions may need to be defined. For example, if a more severe alarming situation occurs, this may overrule a previous nurse call by sending a new nurse call with higher priority. Because the LRS and BRS components allow for defining a priority ordered list of event-based processing queries, such flexibility can be incorporated into the system.

The presented system has a few drawbacks and limitations. In the current architecture, no loss of connectivity to the cloud is assumed. To solve this, a buffer component may be incorporated between every LRS-BRS connection to cope with connectivity losses. This component may run on the same node as the LRS component, or may even be incorporated in the LRS by updating the reasoning service presented in Section 5.3.1. In case of connectivity losses, outgoing events will be buffered, and will be sent in order of arrival to the observing BRS components as soon as the connection is reestablished. Moreover, the current system is not capable of detecting false alarms. For example, severe noise may be present in the sensor observations, causing a series of light intensity observations to be quite stable with a random outlier above the threshold. In the current system, a symptom and potential fault will be generated. Future research should investigate how the system and the queries can be made more intelligent to be able to detect outliers and false alarms. Also when light intensity or sound values are gradually increasing, they can be fluctuating around the threshold at some point. Similarly, the system should be made more intelligent to avoid generating random symptoms and alarms depending on the observed noisy sensor value.

The presented evaluation has been performed in ideal simulation conditions: there was only small Gaussian noise added to the sensor observations, and only one patient and OBU were considered. As explained in the previous paragraph, more severe and unpredictable noise may cause issues that need to be solved in future research. The impact of simulating with only one patient in the used architectural set-up is small locally, as a consequence of combining Fog computing with cascading reasoning: an RSPS is only responsible for one patient, and an LRS for all patients in a single hospital room. The impact on the BRS will be larger, but investigating the scaling of a cloud component with an oNCS has already been investigated in previous research [62].

The concept of cascading reasoning and Fog computing has a big advantage with respect to the amount of events sent to the different components. For the evaluation scenarios, this is clearly shown in Table 4. These scenarios highlight specific situations. However, it is explicitly interesting to investigate a real-life situation. Assuming the evaluation set-up of Section 6 with a single-person room, 7920 observation events are generated per hour by each OBU: 3600 light intensity observations, 3600 sound observations and 720 BLE tag observations, assuming that only the patient is present in the room. Assuming a small hospital with 20 single-person rooms, 158,400 events are generated per hour by all OBUs. In a real-life hospital setting, multiple other sensors will also be producing observations, making the actual value of events per hour a multiple of this value. In another set-up with only one BRS component performing all reasoning and processing, all of these events will arrive at this BRS. This puts a high burden on the BRS. Such a set-up is comparable to many of the recent cloud-based AAL solutions addressed in Section 2.3. In the cascading set-up, the amount of events arriving at the BRS will be a few orders of magnitude smaller, ranging from a few tens to a few hundreds per hour. At the LRS, the amount of events will be another order of magnitude larger, but still way smaller than the original amount of events received by the RSPS. This cascading set-up has many advantages. The events do not need to be processed any longer by a single component, avoiding a single point of failure. Events that can be processed locally will be processed locally, improving the autonomy of the system components. Moreover, the network traffic is reduced, decreasing the transmission cost and increasing the available bandwidth. The back-end and network resources are saved for situations with higher urgency and priority. This all improves the overall responsiveness, throughput and Quality-of-Service of the system. Note that some of the current AAL solutions presented in Section 2.3 also partly have some of these advantages. However, recall that none of these solutions combines stream and cascading reasoning. As a consequence, they are not able to perform real-time analysis on the data streams to make time-critical decisions, which is one of the main objectives of the presented system.

The advantages mentioned in the preceding paragraph can all be relevant for several use cases, inside and outside healthcare. Referring to the smart healthcare requirements addressed in Section 1.1, the presented cascading reasoning platform offers a solution to them. Personalized decision-making is possible in a time-critical way, as shown by the performance evaluation results. Given the example of the presented PoC use case, alarming situations for a patient can be detected locally. While this detection is processed by the BRS to call a nurse to the room, local and edge components can already take automatic action to partially solve the alarming situation, e.g., by dimming the lights for a concussion patient. Moreover, the architecture can cope with a limited amount of resources. Less expensive hardware should be invested in for local and edge components, while investment in more expensive high-level devices for the BRS components can be limited. Furthermore, the generic architecture has a high degree of configurability, allowing for flexible privacy management. For example, if required, sensitive data can already be processed locally, so that it does not need to be transmitted over the backbone network to the back-end. By fulfilling these requirements and offering a solution to many of the issues that exist in ambient-intelligent healthcare, the presented cascading reasoning platform and architecture have the potential to be incorporated in real-life healthcare settings.

## 9. Conclusions and Future Work

In this paper, a cascading reasoning framework is proposed, which can be used to support responsive ambient-intelligent healthcare interventions. A generic cascading architecture is presented that allows for constructing a pipeline of reasoning components, which can be hosted locally, in the edge of the network, and in the cloud, corresponding to the Fog computing principles. The architecture is implemented and evaluated on a pervasive health use case situated in hospital care, where medically diagnosed patients are constantly monitored. A performance evaluation has shown that the total system latency is lower than 5 s in almost all cases, allowing for fast intervention by a nurse in case of an alarming situation. It is discussed how the cascading reasoning platform solves existing issues in smart healthcare, by offering the possibility to perform personalized time-critical decision-making, by enabling the usage of heterogeneous devices with limited resources, and by allowing for flexible privacy management. Additional advantages include reduced network traffic, saving of back-end resources for high priority situations, improved responsiveness and autonomy, and removal of a single point of failure. Future work offers some interesting pathways. First, it should be researched how to deal with connectivity losses and noisy sensor observations, which are current system drawbacks. Second, a large scale evaluation of the platform should be performed with multiple devices and different healthcare scenarios in realistic conditions. To this end, data collection of representative patient profiles and healthcare scenarios is currently ongoing. Third, the framework can be extended to adaptively distribute a federated cascading reasoning system across the IoT fog, also taking into account the scaling of the full system.

## Figures and Tables

**Table 1:** Reasoning support in state-of-the-art RDF Stream Processing (RSP) engines.

	Background Knowledge	Reasoning Capabilities
C-SPARQL	Yes	RDFS
SPARQLStream	Yes	None
EP-SPARQL	Yes	RDFS (in Prolog)
CQELS	Yes	None
Sparkwave	Yes	RDFS subset
INSTANS	No	None

**Table 2:** Overview of the properties of the most prevalent state-of-the-art Ambient Assisted Living (AAL) frameworks and solutions, compared to the solution presented in this work.

			Cascading	Stream	Fog Computing
	Semantics	Reasoning Expressivity	Reasoning	Reasoning	Principles
This work	✔	OWL DL & OWL RL	✔	✔	✔
R-Core [41]		rule-based contextual reasoning			✔
Valencia et al. [42]		case-based reasoning			
Nguyen et al. [43]		multi-objective reasoning			
IoT-SIM [44]	✔	ontology-based *(unspecified)*			✔
SeDan [45]	✔	plausible reasoning			
OCarePlatform [46]	✔	OWL DL			
Lassiera et al. [47]	✔	ontology-based *(unspecified)*			✔
Kuijs et al. [48]	✔	OWL DL			
CoCaMAAL [49]	✔	ontology-based *(unspecified)*			
ERMHAN [50]	✔	OWL DL & rule-based	✔		✔
PERSONA [51]	✔	rule-based & supervised learning			

**Table:** **(a)** Scenario 1

Time	Light	BLE
0	125	B,S
5	125	B,S
10	125	B,S
15	125	B,S
20	125	B,S
25	125	B,S
30	400	B,S
35	400	B,S
40	125	B,S
45	125	B,S
50	125	B,S
55	125	B,S

**Table:** **(b)** Scenario 2

Time	Light	BLE
0	125	B
5	125	B
10	125	B
15	125	B
20	125	B
25	125	B
30	400	B
35	400	B
40	400	B
45	400	B
50	400	B
55	400	B
60	400	B,S
65	125	B,S
70	125	B,S
75	125	B,S

**Table 4:** Average amount of incoming RDF events on the RSP Service (RSPS), Local Reasoning Service (LRS) and Back-end Reasoning Service (BRS) component for each scenario (averaged over all scenario runs).

Scenario	RSPS	LRS	BRS
baseline	131.94	0	0
1	143.86	2.44	0
2	179.68	7.56	1

## Abbreviations

The following abbreviations are used in this manuscript:

AAL	Ambient Assisted Living
ASP	Answer Set Programming
BAN	Body Area Network
BLE	Bluetooth Low Energy
BRS	Back-end Reasoning Service
C-SPARQL	Continuous SPARQL
CEP	Complex Event Processing
CPU	Central Processing Unit
DL	Description Logic
DSMS	Data Stream Management System
EHR	Electronic Health Record
ELE	Enhanced Living Environments
IoT	Internet-of-Things
LRS	Local Reasoning Service
MHz	MegaHertz
OBDA	Ontology-Based Data Access
OBU	Observation Unit
oNCS	ontology-based Nurse Call System
OWL	Web Ontology Language
PoC	Proof-of-Concept
RAM	Random Access Memory
RDF	Resource Description Framework
RDFS	RDF Schema
RSP	RDF Stream Processing
RSPS	RDF Stream Processing Service
SPARQL	Sparql Protocol And RDF Query Language
SSN	Semantic Sensor Network
W3C	World Wide Web Consortium
WSN	Wireless Sensor Network

## References

[1] Burgelman J.C., Punie Y. (2006). Information, society and technology. True Visions.

[2] Al-Fuqaha A., Guizani M., Mohammadi M., Aledhari M., Ayyash M. (2015). Internet of Things: A survey on enabling technologies, protocols, and applications. IEEE Commun. Surv. Tutor..

[3] Perera C., Zaslavsky A., Christen P., Georgakopoulos D. (2014). Context aware computing for the internet of things: A survey. IEEE Commun. Surv. Tutor..

[4] Ongenae F., Famaey J., Verstichel S., De Zutter S., Latré S., Ackaert A., Verhoeve P., De Turck F. (2014). Ambient-aware continuous care through semantic context dissemination. BMC Med. Inform. Decis. Mak..

[5] Internet of Medical Things, Forecast to 2021. https://store.frost.com/internet-of-medical-things-forecast-to-2021.html.

[6] Aggarwal C.C., Ashish N., Sheth A. (2013). The internet of things: A survey from the data-centric perspective. Managing and Mining Sensor Data.

[7] Barnaghi P., Wang W., Henson C., Taylor K. (2012). Semantics for the Internet of Things: Early progress and back to the future. Int. J. Semant. Web Inf. Syst..

[8] Gruber T.R. (1993). A translation approach to portable ontology specifications. Knowl. Acquis..

[9] Compton M., Barnaghi P., Bermudez L., GarcíA-Castro R., Corcho O., Cox S., Graybeal J., Hauswirth M., Henson C., Herzog A. (2012). The SSN ontology of the W3C Semantic Sensor Network Incubator Group. Web Semant..

[10] SNOMED. https://www.snomed.org/snomed-ct.

[11] FHIR. http://hl7.org/fhir/index.html.

[12] Bizer C., Heath T., Berners-Lee T. (2011). Linked data: The story so far. Semantic Services, Interoperability and Web Applications: Emerging Concepts.

[13] Glimm B., Horrocks I., Motik B., Stoilos G., Wang Z. (2014). HermiT: An OWL 2 reasoner. J. Autom. Reason..

[14] Nenov Y., Piro R., Motik B., Horrocks I., Wu Z., Banerjee J. (2015). RDFox: A highly-scalable RDF store. Proceedings of the 2015 International Semantic Web Conference (ISWC 2015).

[15] Dell’Aglio D., Della Valle E., van Harmelen F., Bernstein A. (2017). Stream reasoning: A survey and outlook. Data Sci..

[16] Motik B., Grau B.C., Horrocks I., Wu Z., Fokoue A., Lutz C. (2009). OWL 2 web ontology language profiles. W3C Recomm..

[17] Sahi M.A., Abbas H., Saleem K., Yang X., Derhab A., Orgun M.A., Iqbal W., Rashid I., Yaseen A. (2018). Privacy Preservation in e-Healthcare Environments: State of the Art and Future Directions. IEEE Access.

[18] Su X., Li P., Riekki J., Liu X., Kiljander J., Soininen J.P., Prehofer C., Flores H., Li Y. Distribution of Semantic Reasoning on the Edge of Internet of Things. Proceedings of the 2018 IEEE International Conference on Pervasive Computing and Communications (PerCom).

[19] Margara A., Urbani J., Van Harmelen F., Bal H. (2014). Streaming the web: Reasoning over dynamic data. Web Semant..

[20] Su X., Gilman E., Wetz P., Riekki J., Zuo Y., Leppänen T. Stream reasoning for the Internet of Things: Challenges and gap analysis. Proceedings of the 6th International Conference on Web Intelligence, Mining and Semantics (WIMS 2016 ).

[21] Barbieri D.F., Braga D., Ceri S., Della Valle E., Grossniklaus M. (2010). C-SPARQL: A continuous query language for RDF data streams. Int. J. Semant. Comput..

[22] Le-Phuoc D., Dao-Tran M., Parreira J.X., Hauswirth M. (2011). A native and adaptive approach for unified processing of linked streams and linked data. Proceedings of the 2011 International Semantic Web Conference (ISWC 2011).

[23] Anicic D., Fodor P., Rudolph S., Stojanovic N. (2011). EP-SPARQL: A unified language for event processing and stream reasoning. Proceedings of the International World Wide Web Conference (WWW 2011).

[24] Calbimonte J.P., Corcho O., Gray A.J. Enabling ontology-based access to streaming data sources. Proceedings of the 9th International Semantic Web Conference (ISWC 2010).

[25] Komazec S., Cerri D., Fensel D. (2012). Sparkwave: Continuous schema-enhanced pattern matching over RDF data streams. Proceedings of the 6th ACM International Conference on Distributed Event-Based Systems (DEBS 2012).

[26] Rinne M., Nuutila E., Törmä S. INSTANS: High-performance event processing with standard RDF and SPARQL. Proceedings of the 11th International Semantic Web Conference (ISWC 2012).

[27] Forgy C.L. (1982). Rete: A fast algorithm for the many pattern/many object pattern match problem. Artif. Intell..

[28] Germano S., Pham T.L., Mileo A. (2015). Web stream reasoning in practice: On the expressivity vs. scalability tradeoff. Proceedings of the 9th International Conference on Web Reasoning and Rule Systems (RR 2015).

[29] Stuckenschmidt H., Ceri S., Della Valle E., Van Harmelen F. (2010). Towards expressive stream reasoning. Proceedings of the Dagstuhl Seminar 10042.

[30] Dastjerdi A.V., Gupta H., Calheiros R.N., Ghosh S.K., Buyya R. (2016). Fog computing: Principles, architectures, and applications. Internet of Things.

[31] Mahmud R., Kotagiri R., Buyya R. (2018). Fog computing: A taxonomy, survey and future directions. Internet of Everything.

[32] Gedeon J., Heuschkel J., Wang L., Mühlhäuser M. (2018). Fog Computing: Current Research and Future Challenges. KuVS-Fachgespräch Fog Comput..

[33] Skarlat O., Bachmann K., Schulte S. (2018). FogFrame: IoT Service Deployment and Execution in the Fog. KuVS-Fachgespräch Fog Comput..

[34] Gyrard A., Datta S.K., Bonnet C., Boudaoud K. A semantic engine for Internet of Things: Cloud, mobile devices and gateways. Proceedings of the Ninth International Conference on Innovative Mobile and Internet Services in Ubiquitous Computing (IMIS 2015).

[35] Sedira Y.A., Tommasini R., Della Valle E. MobileWave: Publishing RDF Streams from SmartPhones. Proceedings of the 16th International Semantic Web Conference (ISWC 2017).

[36] Van Woensel W., Abidi S.S.R. Optimizing Semantic Reasoning on Memory-Constrained Platforms Using the RETE Algorithm. Proceedings of the 2018 Extended Semantic Web Conference (ESWC 2018).

[37] Charpenay V., Käbisch S., Kosch H. Towards a Binary Object Notation for RDF. Proceedings of the 2018 Extended Semantic Web Conference (ESWC 2018).

[38] Garcia N.M., Rodrigues J.J.P. (2015). Ambient Assisted Living.

[39] Goleva R.I., Ganchev I., Dobre C., Garcia N., Valderrama C. (2017). Enhanced Living Environments: From Models to Technologies.

[40] Goleva R.I., Garcia N., Mavromoustakis C.X., Dobre C., Dobre C., Mavromoustakis C.X., Garcia N., Goleva R.I., Mastorakis G. (2016). AAL and ELE Platform Architecture. Ambient Assisted Living and Enhanced Living Environments: Principles, Technologies and Control.

[41] Moawad A. (2016). Towards ambient intelligent applications using models@run.time and machine learning for context-awareness. PhD thesis.

[42] Valencia X.B., Torres D.B., Rodriguez C.P., Peluffo-Ordóñez D.H., Becerra M.A., Castro-Ospina A.E. (2018). Case-Based Reasoning Systems for Medical Applications with Improved Adaptation and Recovery Stages. Proceedings of the 2018 International Conference on Bioinformatics and Biomedical Engineering (IWBBIO 2018).

[43] Nguyen C.M., Sebastiani R., Giorgini P., Mylopoulos J. (2018). Multi-objective reasoning with constrained goal models. Requir. Eng..

[44] Jabbar S., Ullah F., Khalid S., Khan M., Han K. (2017). Semantic interoperability in heterogeneous IoT infrastructure for healthcare. Wirel. Commun. Mob. Comput..

[45] Mohammadhassanzadeh H., Abidi S.R., Shah M.S., Karamollahi M., Abidi S.S.R. SeDAn: A Plausible Reasoning Approach for Semantics-based Data Analytics in Healthcare. Proceedings of the 7th International Workshop on Artificial Intelligence in Medical Applications (WAIAH 2017).

[46] De Backere F., Bonte P., Verstichel S., Ongenae F., De Turck F. (2017). The OCarePlatform: A context-aware system to support independent living. Comput. Methods Programs Biomed..

[47] Lasierra N., Alesanco A., Garcia J. (2014). Designing an architecture for monitoring patients at home: Ontologies and web services for clinical and technical management integration. IEEE J. Biomed. Health Inform..

[48] Kuijs H., Rosencrantz C., Reich C. A context-aware, intelligent and flexible ambient assisted living platform architecture. Proceedings of the 6th International Conference on Cloud Computing, GRIDs, and Virtualization.

[49] Forkan A., Khalil I., Tari Z. (2014). CoCaMAAL: A cloud-oriented context-aware middleware in ambient assisted living. Future Gener. Comput. Syst..

[50] Paganelli F., Giuli D. (2011). An ontology-based system for context-aware and configurable services to support home-based continuous care. IEEE Trans. Inf. Technol. Biomed..

[51] Amoretti M., Copelli S., Wientapper F., Furfari F., Lenzi S., Chessa S. (2013). Sensor data fusion for activity monitoring in the PERSONA ambient assisted living project. J. Ambient Intell. Humaniz. Comput..

[52] Poggi A., Lembo D., Calvanese D., De Giacomo G., Lenzerini M., Rosati R. (2008). Linking data to ontologies. Journal on Data Semantics X.

[53] Designed Continuous Care Ontology. https://github.com/IBCNServices/cascading-reasoning-framework.

[54] ACCIO Ontology. https://github.com/IBCNServices/Accio-Ontology/tree/gh-pages.

[55] Ongenae F., Duysburgh P., Sulmon N., Verstraete M., Bleumers L., De Zutter S., Verstichel S., Ackaert A., Jacobs A., De Turck F. (2014). An ontology co-design method for the co-creation of a continuous care ontology. Appl. Ontol..

[56] Daniele L., den Hartog F., Roes J. (2015). Created in close interaction with the industry: The smart appliances reference (SAREF) ontology. Proceedings of the 7th International Formal Ontologies Meet Industries Workshop (FOMI 2015).

[57] Calvanese D., Cogrel B., Komla-Ebri S., Kontchakov R., Lanti D., Rezk M., Rodriguez-Muro M., Xiao G. (2017). Ontop: Answering SPARQL queries over relational databases. Semant. Web.

[58] Stardog. https://www.stardog.com.

[59] RSP Service Interface for C-SPARQL. https://github.com/streamreasoning/rsp-services-csparql/.

[60] OWL 2 RL Profile. https://www.w3.org/TR/owl2-profiles/#OWL_2_RL.

[61] Nelis J., Verschueren T., Verslype D., Develder C. Dyamand: Dynamic, adaptive management of networks and devices. Proceedings of the 37th Annual IEEE Conference on Local Computer Networks (LCN 2012).

[62] Ongenae F., Myny D., Dhaene T., Defloor T., Van Goubergen D., Verhoeve P., Decruyenaere J., De Turck F. (2011). An ontology-based nurse call management system (oNCS) with probabilistic priority assessment. BMC Health Serv. Res..

[63] Specifications of an Intel NUC, Model D54250WYKH. https://ark.intel.com/products/81164/Intel-NUC-Kit-D54250WYKH.

[64] Documentation of the Imec iLab.t Testbeds Virtual Wall. https://doc.ilabt.imec.be/ilabt-documentation/.

[65] Bonte P., Ongenae F., Schaballie J., De Meester B., Arndt D., Dereuddre W., Bhatti J., Verstichel S., Verborgh R., Van de Walle R. Evaluation and optimized usage of OWL 2 reasoners in an event-based eHealth context. Proceedings of the 4th OWL Reasoner Evaluation Competition (ORE 2015).

